# Supplementary material for: A Likelihood Approach for Real-Time Calibration of Stochastic Compartmental Epidemic Models
Source: PLoS Comput Biol. 2017 Jan 17;13(1):e1005257. doi: 10.1371/journal.pcbi.1005257 (PMC5240920; doi:10.1371/journal.pcbi.1005257)
Supplement: S1 File — (TAR.GZ) [file pcbi.1005257.s014.tar.gz › HSPH_Online-SI-Revision/MSS10/n1-severe/n1-severe_NEW_table4.pdf]

1	R0	{1.51081, 13.7078, 1.}	{1.25451, 3.16049, 0.02}	{{1.22544, 2.5364, 0.}, {1.28596, 3.98513, 0.}}	{16.9644, 76.9439, 98.}	inf	60.	11.9558	{4.27461, 22.0554}	80.0736	0.78042	newly	28.	20.5	{6., 40.}	45.7143	0.302721	Reff	0.861162	0.775379	{0.752835, 0.792489}	10.3275	ar	656.	414.22	{378., 461.}	36.8567
2	R0	{1.73182, 11.3873, 3.}	{1.45659, 4.19538, 0.05}	{{1.44485, 3.83841, 0.}, {1.47608, 4.94504, 0.}}	{15.8925, 63.1574, 98.3333}	inf	73.	19.7673	{7.00559, 31.7284}	72.9216	0.634724	newly	43.	27.55	{10., 46.}	41.6047	0.28978	Reff	0.82781	0.773804	{0.75173, 0.79401}	6.55426	ar	653.	516.72	{463., 570.}	20.8698
3	R0	{1.50449, 10.3474, 0.1}	{1.41641, 3.8778, 0.03}	{{1.25474, 3.26445, 0.}, {1.47608, 4.06661, 0.}}	{5.89481, 62.5239, 126.}	inf	61.	21.4635	{8.922, 32.6515}	64.8139	0.505297	newly	34.	33.41	{14., 53.}	36.8529	0.195635	Reff	0.825965	0.833445	{0.733517, 0.877986}	5.32186	ar	651.	484.73	{423., 546.}	25.5561
4	R0	{1.63493, 9.1955, 0.1}	{1.79111, 10.5582, 0.12}	{{1.71608, 9.75208, 0.}, {1.83592, 11.1369, 0.}}	{9.84213, 17.4016, 204.}	inf	51.	76.5384	{50.1632, 92.3732}	52.0074	0.174971	newly	40.	46.27	{32., 58.}	23.175	0.0908234	Reff	0.734084	0.757515	{0.734055, 0.778791}	3.60871	ar	663.	708.76	{656., 751.}	8.16591
5	R0	{1.53822, 8.05553, 0.1}	{1.76718, 14.4263, 2.44}	{{1.56097, 8.77207, 2.}, {1.95859, 19.3194, 3.}}	{14.8845, 79.0851, 2340.}	inf	25.	93.4169	{47.6688, 115.456}	273.668	0.551039	newly	19.	43.51	{31., 55.}	129.	0.34942	Reff	0.772186	0.763375	{0.722466, 0.821873}	4.81999	ar	621.	715.12	{602., 784.}	16.3671
6	R0	{1.69374, 17.0793, 0.1}	{1.74747, 19.0506, 0.97}	{{1.75588, 19.2972, 1.}, {1.75588, 19.2972, 1.}}	{3.94522, 13.6506, 876.}	inf	95.	92.3101	{83.2553, 103.907}	7.9566	0.0394185	newly	35.	33.36	{25., 41.}	14.8	0.0691836	Reff	0.98745	1.023	{1.0042, 1.03944}	3.85582	ar	662.	692.12	{646., 743.}	6.40785
7	R0	{1.42179, 11.8031, 2.}	{1.54174, 13.1783, 2.}	{{1.54363, 13.2419, 2.}, {1.54363, 13.2419, 2.}}	{8.53089, 12.4849, 0.}	inf	48.	51.9789	{44.8365, 60.9234}	13.0704	0.0559383	newly	29.	26.04	{19., 34.}	17.931	0.0897603	Reff	0.796202	0.857162	{0.844266, 0.868554}	7.65632	ar	517.	593.49	{534., 645.}	15.2282
8	R0	{1.46683, 6.95875, 0.1}	{1.67562, 7.30904, 1.16}	{{1.67405, 7.28757, 1.}, {1.68297, 7.5059, 2.}}	{14.2343, 5.26361, 1060.}	inf	30.	33.0348	{24.2068, 41.4738}	20.0623	0.0818004	newly	30.	26.47	{18., 36.}	20.5667	0.104441	Reff	0.545661	0.618244	{0.602562, 0.632883}	13.3019	ar	653.	679.39	{651., 707.}	4.33231
9	R0	{1.34525, 11.505, 1.}	{1.25252, 3.06335, 0.03}	{{1.22544, 2.5364, 0.}, {1.25474, 3.26445, 0.}}	{7.98346, 74.3189, 97.}	inf	49.	11.4868	{1.68241, 21.3131}	76.5575	0.772949	newly	38.	22.19	{2., 44.}	52.3947	0.432365	Reff	0.881139	0.867081	{0.844527, 0.876207}	2.801	ar	532.	356.27	{299., 450.}	33.4756
10	R0	{1.77958, 14.372, 0.1}	{1.66055, 15.2961, 2.}	{{1.66055, 15.2961, 2.}, {1.66055, 15.2961, 2.}}	{6.6888, 6.43017, 1900.}	inf	58.	80.7057	{72.5217, 90.7051}	39.1478	0.142056	newly	28.	34.1	{26., 42.}	25.0714	0.0939976	Reff	0.726069	0.639799	{0.62305, 0.652463}	11.8817	ar	671.	710.68	{681., 742.}	5.99702
11	R0	{1.60334, 19.9548, 1.}	{1.42059, 17.1299, 2.99}	{{1.42136, 17.2398, 3.}, {1.42136, 17.2398, 3.}}	{11.3982, 14.1565, 199.}	inf	78.	66.0012	{56.7643, 73.4024}	15.4377	0.0754966	newly	24.	26.07	{21., 32.}	15.375	0.0620306	Reff	0.928334	0.839539	{0.829332, 0.84994}	9.56501	ar	617.	572.11	{519., 623.}	7.99514
12	R0	{1.68623, 7.58585, 0.1}	{1.51206, 4.1984, 0.56}	{{1.47488, 4.02511, 0.}, {1.53501, 4.79544, 3.}}	{10.3289, 44.6549, 626.}	inf	21.	9.91783	{3.71175, 14.9376}	52.7722	0.379697	newly	21.	12.17	{4., 19.}	43.	0.301984	Reff	0.696413	0.641214	{0.621894, 0.656467}	7.92622	ar	640.	592.01	{574., 610.}	7.71406
13	R0	{1.82689, 18.0022, 1.}	{1.86583, 12.6015, 0.}	{{1.44485, 6.00596, 0.}, {2.23915, 18.4504, 0.}}	{21.7888, 32.6392, 100.}	inf	97.	85.8968	{14.2736, 148.696}	60.9655	0.402554	newly	31.	37.87	{13., 57.}	51.8387	0.217442	Reff	0.811139	0.825394	{0.739369, 0.905572}	8.90328	ar	673.	687.28	{494., 848.}	21.9911
14	R0	{1.75911, 9.16883, 0.1}	{1.48085, 4.54711, 0.31}	{{1.44762, 4.00571, 0.}, {1.53501, 6.00596, 1.}}	{15.8614, 50.9535, 378.}	inf	49.	23.2336	{9.50612, 37.6397}	56.5731	0.417104	newly	35.	27.65	{10., 44.}	33.9714	0.210816	Reff	0.809191	0.718768	{0.697057, 0.736614}	11.1744	ar	661.	561.38	{510., 626.}	15.2617
15	R0	{1.46168, 11.3859, 2.}	{1.43293, 7.01257, 1.69}	{{1.42446, 6.00596, 0.}, {1.44485, 7.81552, 3.}}	{1.96716, 38.41, 71.5}	inf	42.	15.7172	{8.3149, 23.5511}	62.5781	0.462535	newly	28.	13.51	{7., 19.}	51.75	0.350883	Reff	0.69576	0.719749	{0.704768, 0.734826}	3.44925	ar	556.	533.66	{505., 561.}	5.17986
16	R0	{1.53577, 11.7581, 1.}	{1.44483, 6.04298, 0.09}	{{1.44485, 6.00596, 0.}, {1.44485, 6.00596, 0.}}	{6.19204, 48.6058, 93.}	inf	70.	31.9463	{20.186, 42.7469}	54.3624	0.359795	newly	40.	32.04	{18., 43.}	24.1	0.132963	Reff	0.7771	0.785935	{0.768344, 0.801827}	1.68416	ar	625.	537.34	{493., 588.}	14.1536
17	R0	{1.70698, 9.72924, 0.1}	{1.80385, 10.5, 1.6}	{{1.71337, 8.80249, 1.}, {2.01498, 14.4607, 3.}}	{5.67511, 21.2572, 1500.}	inf	45.	59.9666	{40.3068, 89.0378}	37.7086	0.125421	newly	22.	34.48	{25., 42.}	56.9091	0.189089	Reff	0.626462	0.632574	{0.609695, 0.658807}	2.49173	ar	675.	721.27	{674., 788.}	7.16
18	R0	{1.5973, 14.931, 0.1}	{1.25858, 3.80905, 0.33}	{{1.21159, 3.07157, 0.}, {1.28596, 4.21286, 2.}}	{21.2059, 74.489, 386.}	inf	48.	14.0063	{3.42004, 25.4051}	70.8201	0.626052	newly	24.	23.77	{5., 40.}	44.125	0.249794	Reff	1.13089	0.93382	{0.899043, 0.962271}	17.426	ar	632.	338.29	{257., 445.}	46.4731
19	R0	{1.87468, 17.004, 1.}	{1.55644, 10.5556, 1.}	{{1.55644, 10.5556, 1.}, {1.55644, 10.5556, 1.}}	{16.9755, 37.9231, 0.}	inf	76.	40.4021	{32.1944, 47.3729}	46.8394	0.278509	newly	35.	24.6	{19., 30.}	29.7143	0.160267	Reff	0.783616	0.705999	{0.695042, 0.71702}	9.90495	ar	692.	620.08	{587., 659.}	10.3931
20	R0	{1.84715, 12.531, 0.1}	{1.5589, 11.5367, 1.}	{{1.52414, 9.52864, 1.}, {1.58973, 13.3174, 1.}}	{15.6049, 14.587, 900.}	inf	47.	33.3334	{21.2386, 45.561}	30.0398	0.17193	newly	27.	18.85	{8., 31.}	36.7037	0.227432	Reff	0.718541	0.627981	{0.59288, 0.659352}	12.6033	ar	661.	644.51	{613., 678.}	3.59002
21	R0	{1.75938, 13.5959, 2.}	{1.38898, 6.38702, 0.21}	{{1.38815, 5.7404, 0.}, {1.40191, 6.71734, 1.}}	{21.0531, 53.0224, 89.5}	inf	73.	26.328	{16.4347, 34.9799}	63.9342	0.470808	newly	35.	25.64	{16., 35.}	29.6	0.172686	Reff	0.932471	0.800943	{0.786497, 0.817039}	14.1054	ar	620.	498.13	{441., 552.}	19.6565
22	R0	{1.63456, 10.7469, 0.1}	{1.55077, 5.50976, 0.81}	{{1.47608, 4.02511, 0.}, {1.58886, 7.29751, 3.}}	{5.12601, 48.7316, 804.}	inf	44.	15.4361	{5.1717, 26.4825}	64.918	0.537348	newly	29.	15.12	{5., 24.}	47.8621	0.352309	Reff	0.626036	0.638025	{0.620205, 0.651491}	2.37016	ar	654.	611.75	{582., 646.}	6.52446
23	R0	{1.62702, 19.1392, 1.}	{1.38972, 4.58342, 0.48}	{{1.28596, 3.98513, 0.}, {1.44762, 5.7404, 1.}}	{14.5849, 76.0522, 60.}	inf	90.	15.4085	{6.40227, 24.5111}	82.8794	0.82096	newly	31.	20.19	{9., 34.}	41.5161	0.272762	Reff	0.810256	0.795661	{0.740643, 0.841933}	3.88317	ar	600.	476.07	{430., 529.}	20.655
24	R0	{1.70466, 12.1202, 2.}	{1.50186, 3.88513, 0.02}	{{1.47488, 3.83841, 0.}, {1.53501, 4.06661, 0.}}	{11.8968, 67.945, 99.}	inf	45.	12.9469	{3.94722, 20.5561}	71.2292	0.60506	newly	23.	18.74	{5., 32.}	36.6087	0.229275	Reff	0.758574	0.716424	{0.694995, 0.738435}	5.88754	ar	613.	552.61	{519., 588.}	10.0408
25	R0	{1.61735, 11.3169, 0.1}	{1.55987, 3.99135, 0.07}	{{1.53501, 3.37285, 0.}, {1.65456, 4.02511, 0.}}	{4.55403, 64.7311, 160.}	inf	43.	12.4921	{3.69482, 21.6288}	70.9487	0.627569	newly	31.	16.25	{4., 28.}	50.2903	0.394469	Reff	0.608124	0.634076	{0.610169, 0.672871}	4.44903	ar	681.	613.85	{588., 647.}	9.8605
26	R0	{1.56977, 12.3035, 2.}	{1.47574, 6.80706, 0.8}	{{1.28596, 3.83841, 0.}, {1.79724, 16.8903, 3.}}	{11.6176, 58.8846, 78.}	inf	36.	37.6549	{7.65934, 120.541}	83.9752	0.376629	newly	18.	29.12	{10., 50.}	79.4444	0.250052	Reff	0.846106	0.786371	{0.720181, 0.824776}	7.16018	ar	594.	541.92	{450., 756.}	18.5724
27	R0	{1.46317, 5.8298, 0.1}	{1.72667, 10.246, 1.3}	{{1.53501, 4.02511, 0.}, {1.82711, 13.6186, 2.}}	{18.0092, 97.9324, 1270.}	inf	14.	71.312	{7.13463, 108.849}	427.964	0.642485	newly	14.	36.63	{11., 54.}	173.071	0.418331	Reff	0.626237	0.634102	{0.608629, 0.66717}	3.12712	ar	586.	709.45	{578., 791.}	21.7048
28	R0	{1.70002, 14.9806, 1.}	{1.78827, 12.5828, 1.93}	{{1.39162, 1.89883, 0.}, {1.93288, 15.3647, 3.}}	{13.1397, 20.0839, 121.}	inf	101.	100.063	{21.7688, 136.686}	35.6256	0.223359	newly	48.	51.16	{35., 64.}	19.375	0.0862994	Reff	0.85511	0.892231	{0.825526, 0.941955}	6.07826	ar	652.	697.6	{502., 790.}	16.3742
29	R0	{1.52729, 16.1014, 3.}	{1.38828, 6.73344, 0.28}	{{1.38815, 6.71734, 0.}, {1.38815, 6.71734, 1.}}	{9.10206, 58.181, 90.6667}	inf	46.	17.8071	{9.86013, 25.015}	61.289	0.442381	newly	17.	16.27	{8., 24.}	28.7647	0.144888	Reff	0.781972	0.749927	{0.739543, 0.760196}	4.098	ar	592.	504.11	{467., 540.}	14.8733
30	R0	{1.72795, 13.5635, 1.}	{1.5137, 15.8672, 4.96}	{{1.51444, 16.0011, 5.}, {1.51444, 16.0011, 5.}}	{12.3993, 18.6002, 396.}	inf	61.	59.7891	{53.9696, 66.431}	6.77408	0.039469	newly	28.	23.41	{17., 29.}	18.8214	0.0997247	Reff	0.661805	0.581536	{0.571752, 0.590644}	12.1288	ar	668.	681.68	{655., 705.}	2.91916
31	R0	{1.51591, 18.1544, 3.}	{1.34647, 8.41762, 3.02}	{{1.34668, 8.42466, 3.}, {1.34668, 8.42466, 3.}}	{11.1772, 53.6332, 0.666667}	inf	51.	33.9256	{25.335, 41.133}	33.5562	0.186628	newly	16.	26.99	{18., 34.}	69.4375	0.218864	Reff	0.950476	0.867231	{0.856637, 0.878687}	8.75822	ar	541.	475.07	{417., 542.}	13.1553
32	R0	{1.4282, 10.3534, 0.1}	{1.30566, 4.99313, 0.37}	{{1.28596, 3.98513, 0.}, {1.44709, 12.0436, 2.}}	{8.87168, 56.3299, 434.}	inf	33.	16.9376	{4.07939, 33.5811}	66.6667	0.501121	newly	26.	21.13	{6., 37.}	41.0385	0.256432	Reff	1.03687	0.968169	{0.949831, 0.998083}	6.734	ar	521.	360.64	{249., 531.}	32.9712
33	R0	{1.56693, 11.3972, 0.1}	{1.78639, 11.7314, 3.1}	{{1.6082, 8.12461, 2.}, {2.31174, 17.2836, 4.}}	{14.0059, 29.3068, 3000.}	inf	84.	86.2399	{39.5624, 165.006}	50.5305	0.225802	newly	50.	44.03	{26., 71.}	31.38	0.154417	Reff	0.634607	0.708107	{0.682886, 0.728699}	11.582	ar	695.	710.21	{608., 845.}	11.1324
34	R0	{1.50675, 19.8823, 1.}	{1.37237, 4.91592, 1.02}	{{1.3618, 4.06661, 0.}, {1.41571, 4.98002, 1.}}	{8.91827, 75.2749, 22.}	inf	63.	9.6904	{2.45421, 16.2934}	84.6184	0.893083	newly	29.	12.14	{3., 21.}	58.9655	0.482667	Reff	0.797071	0.799104	{0.784143, 0.823417}	1.45591	ar	587.	452.52	{416., 506.}	22.9097
35	R0	{1.72889, 7.76068, 0.1}	{1.59168, 7.33588, 1.01}	{{1.58886, 7.29751, 1.}, {1.58886, 7.29751, 1.}}	{7.93602, 6.1045, 910.}	inf	43.	31.9884	{23.3283, 39.0213}	28.82	0.154297	newly	34.	25.62	{17., 35.}	28.2941	0.154464	Reff	0.7002	0.662058	{0.649812, 0.674719}	5.44738	ar	650.	633.2	{599., 672.}	4.28308
36	R0	{1.62644, 4.12303, 0.1}	{1.65456, 3.37285, 0.}	{{1.65456, 3.37285, 0.}, {1.65456, 3.37285, 0.}}	{1.72895, 18.1949, 100.}	inf	8.	8.02497	{2.82657, 13.5299}	41.2231	0.20471	newly	9.	11.08	{3., 19.}	55.7778	0.246505	Reff	0.57088	0.580709	{0.571116, 0.58919}	1.80174	ar	652.	660.8	{646., 679.}	1.73313
37	R0	{1.70878, 15.8072, 1.}	{1.44947, 5.64189, 0.06}	{{1.44485, 4.02511, 0.}, {1.454, 6.00596, 0.}}	{15.1751, 64.3081, 102.}	inf	45.	9.33528	{3.16376, 14.4139}	79.2549	0.756467	newly	13.	10.02	{3., 16.}	41.2308	0.26667	Reff	0.772369	0.706862	{0.697152, 0.716493}	8.48126	ar	674.	533.77	{510., 561.}	20.8056
38	R0	{1.58967, 6.80568, 0.1}	{1.58953, 7.31405, 1.02}	{{1.58886, 7.29751, 1.}, {1.58886, 7.29751, 1.}}	{0.0902196, 7.46982, 920.}	inf	25.	29.8807	{21.8593, 37.7659}	25.9256	0.0988393	newly	22.	23.97	{16., 32.}	24.6818	0.103641	Reff	0.661303	0.65348	{0.640684, 0.665084}	1.5416	ar	610.	635.23	{604., 663.}	4.61475
39	R0	{1.344, 8.19498, 0.1}	{1.39259, 7.17493, 0.82}	{{1.35478, 4.98002, 0.}, {1.44476, 10.1243, 2.}}	{3.74113, 22.8063, 828.}	inf	37.	26.7897	{12.8765, 39.0095}	31.482	0.191282	newly	28.	23.36	{13., 33.}	26.9286	0.150237	Reff	0.756672	0.798044	{0.780415, 0.816296}	5.478	ar	551.	501.56	{443., 564.}	10.3884
40	R0	{1.61509, 7.90825, 0.1}	{1.45937, 3.89835, 0.02}	{{1.44762, 3.83841, 0.}, {1.47608, 4.06661, 0.}}	{9.64131, 50.7052, 118.}	inf	33.	15.7632	{6.40484, 24.4386}	52.2326	0.377536	newly	23.	22.42	{9., 35.}	34.1739	0.178134	Reff	0.814005	0.760658	{0.745548, 0.781353}	6.55485	ar	581.	518.42	{480., 566.}	11.1153
41	R0	{1.60017, 5.90605, 0.1}	{1.71159, 8.75472, 0.99}	{{1.71337, 8.80249, 1.}, {1.71337, 8.80249, 1.}}	{7.0443, 48.87, 892.}	inf	23.	37.2882	{30.5983, 45.0577}	63.7591	0.212753	newly	19.	25.59	{19., 32.}	37.8421	0.136544	Reff	0.611265	0.629315	{0.615113, 0.641135}	3.01988	ar	661.	683.33	{654., 710.}	3.88351
42	R0	{1.64436, 15.1337, 1.}	{1.6206, 14.2506, 3.49}	{{1.35478, 6.25107, 0.}, {1.79789, 19.6311, 5.}}	{12.2697, 40.5284, 275.}	inf	68.	74.4816	{19.5419, 113.559}	58.4166	0.287216	newly	31.	32.87	{21., 43.}	23.3226	0.10624	Reff	0.902754	0.871013	{0.79868, 0.929948}	5.62648	ar	640.	623.19	{460., 749.}	17.2016
43	R0	{1.48917, 11.917, 0.1}	{1.39696, 7.93389, 0.08}	{{1.38815, 6.71734, 0.}, {1.39882, 8.26611, 0.}}	{6.19202, 33.4237, 174.}	inf	22.	20.5055	{13.5825, 26.8356}	20.1879	0.0992413	newly	12.	15.99	{10., 22.}	41.4167	0.151354	Reff	0.814576	0.766224	{0.756913, 0.7747}	5.93589	ar	551.	500.13	{461., 552.}	9.49002
44	R0	{1.65577, 7.89317, 1.}	{1.44485, 6.00596, 0.}	{{1.44485, 6.00596, 0.}, {1.44485, 6.00596, 0.}}	{12.7386, 23.9094, 100.}	inf	38.	25.8261	{15.2707, 35.5586}	32.5295	0.192983	newly	25.	25.56	{14., 37.}	27.12	0.125615	Reff	0.819606	0.732789	{0.71863, 0.747894}	10.5926	ar	576.	543.82	{499., 583.}	6.5625
45	R0	{1.6909, 12.3275, 0.1}	{1.54033, 9.04457, 1.}	{{1.54207, 9.08517, 1.}, {1.54207, 9.08517, 1.}}	{8.90495, 26.631, 900.}	inf	39.	31.5474	{23.5125, 40.0267}	20.9453	0.107759	newly	24.	21.59	{14., 30.}	21.2083	0.105994	Reff	0.749069	0.693844	{0.680508, 0.706784}	7.37247	ar	675.	603.42	{575., 636.}	10.6044
46	R0	{1.62351, 16.0698, 2.}	{1.34829, 9.33397, 2.}	{{1.34829, 9.33397, 2.}, {1.34829, 9.33397, 2.}}	{16.952, 41.9161, 0.}	inf	55.	38.8173	{28.727, 48.9219}	30.0056	0.163218	newly	26.	28.41	{20., 38.}	21.0385	0.0847712	Reff	1.091	0.927872	{0.912748, 0.940699}	14.9521	ar	591.	469.65	{390., 541.}	20.5499
47	R0	{1.52372, 4.38599, 0.1}	{1.53072, 4.04025, 0.1}	{{1.47608, 4.02511, 0.}, {1.53501, 4.02511, 0.}}	{1.155, 8.53335, 194.}	inf	12.	11.3585	{3.46984, 19.3334}	36.747	0.195287	newly	15.	14.27	{5., 25.}	38.7333	0.213146	Reff	0.594251	0.597971	{0.580605, 0.611747}	1.76397	ar	618.	624.27	{604., 648.}	1.97249
48	R0	{1.44306, 11.788, 1.}	{1.3839, 6.71528, 0.47}	{{1.36632, 6.71734, 0.}, {1.38815, 6.77341, 2.}}	{4.09933, 43.0329, 101.}	inf	18.	18.3954	{10.9759, 25.6116}	27.6705	0.125579	newly	11.	16.51	{10., 23.}	58.8182	0.194645	Reff	0.793683	0.760578	{0.751301, 0.769978}	4.17111	ar	534.	496.57	{461., 542.}	7.76592
49	R0	{1.63624, 11.3893, 1.}	{1.4602, 5.02206, 0.21}	{{1.44485, 4.00571, 0.}, {1.47608, 6.00596, 0.}}	{10.7588, 55.9054, 107.}	inf	48.	14.811	{5.76579, 24.3152}	69.1437	0.570405	newly	31.	16.78	{6., 28.}	48.0645	0.347327	Reff	0.713401	0.685139	{0.665953, 0.705913}	4.02797	ar	643.	560.43	{533., 596.}	12.8414
50	R0	{1.46113, 11.1322, 1.}	{1.26227, 3.5874, 0.07}	{{1.22544, 3.07157, 0.}, {1.28596, 3.98513, 0.}}	{13.6102, 67.7745, 95.}	inf	30.	12.2427	{3.7041, 19.6537}	59.1912	0.46151	newly	18.	20.02	{5., 34.}	52.	0.247577	Reff	0.936584	0.831517	{0.802997, 0.855013}	11.2182	ar	501.	387.06	{340., 450.}	22.9102
